# Pregnancy in a Transgender Male: A Case Report and Review of the Literature

**DOI:** 10.1155/2022/6246867

**Published:** 2022-06-29

**Authors:** Ayesha Hassan, Jessica Perini, Amna Khan, Apoorva Iyer

**Affiliations:** ^1^Department of Internal Medicine, West Virginia University Hospital, Morgantown, WV, USA; ^2^Department of Endocrinology and Metabolism, West Virginia University Hospital, Morgantown, WV, USA; ^3^Avalon University of Medicine, Youngstown, OH, USA

## Abstract

**Introduction:**

Pregnancy in transgender men is an area of increasing study due to data showing that pregnancy can occur in this population despite the reduction in fertility that generally accompanies treatment with gender-affirming hormone therapies.

**Case:**

In this case, we describe a healthy 21-year-old transgender man who was able to achieve pregnancy without reproductive assistance after stopping his testosterone therapy for 2 months. *Discussion*. Our case is important as it highlights how little is known in regards to gender-affirming hormone therapy on fertility. While testosterone is known to reduce fertility by inducing anovulation and altering ovarian histology, its long-term effects on conception rates and pregnancy are largely unknown. Some studies demonstrate that transgender men, treated with gender-affirming hormone therapy (GAHT), including testosterone, have similar oocyte quantity and quality, as well as similar ovarian reserve, when compared to cisgender women, suggesting that resumption of fertility may be possible after cessation of GAHT. Long-term outcomes for the pregnancy and the offspring of those who have been treated with GAHT are unknown.

**Conclusion:**

Recent studies have shown that pregnancy is possible for transgender men who desire biological children and have received gender-affirming hormonal therapy without fertility-preserving measures. Further research is needed to help determine rates of fertility, the likelihood of recovery of fertility, conception rates, and long-term pregnancy outcomes. Such information would help guide physicians in providing education and counseling to their transgender patients regarding reproductive options.

## 1. Introduction

Gender-affirming hormone therapy (GAHT) in transgender men lowers fertility rates. Nevertheless, many transgender individuals are able to achieve pregnancy. Transgender male pregnancy is a topic that is becoming increasingly prevalent in medical literature. However, many areas pertaining to this topic still require further study.

## 2. Case Presentation

A 21-year-old transgender man who had been prescribed gender-affirming hormone therapy for over two years presented to his follow-up appointment at 17 weeks pregnant. Past medical history included ADHD, asthma, and gender dysphoria. Home medications included albuterol and weekly intramuscular depo-testosterone 100 mg injections, which he had been taking consistently for 2 years prior to conceiving. The gynecological history was unremarkable, and menses had stopped one month after starting testosterone therapy. Testosterone measurements on routine labs performed every 3 to 6 months over the preceding 2 years for the purpose of monitoring testosterone levels had consistently shown levels within the mid-normal range for men.

The patient had stopped his testosterone therapy 2 months prior to conceiving, with the intention of trying to become pregnant. He had a menstrual period one month after discontinuing his testosterone therapy, and the following month a pregnancy test was positive. Pregnancy was achieved through vaginal intercourse with the patient's healthy cismale partner.

Prior to starting GAHT, the patient had been counseled about reduced fertility with hormone therapy and was given a referral to reproductive endocrinology; however, at that time, he had not desired future pregnancy and did not undergo any fertility-preserving measures.

Neither the patient nor his partner received any reproductive assistance, and the patient underwent pregnancy without any significant complications and successfully delivered a healthy baby girl via *C*-section.

## 3. Discussion

Our case report is important because minimal and conflicting data exist regarding fertility, conception rates, and pregnancy in transgender men. Our case report questions the commonly held belief that transgender male pregnancy is highly difficult in people receiving GAHT who have not received fertility preservation. Although many transgender men use gender-affirming hormone therapy to masculinize body parts and align their physical appearance more with their gender identity, the majority retain their uterus and ovaries [1, 2]. One of the effects of GAHT is the cessation of menstrual cycles, which generally occurs within the first few months of medication use. It is not known to what degree fertility can be restored after the cessation of GAHT.

Exogenous testosterone therapy, a mainstay of treatment for transgender men, is associated with reduced fertility rates. Testosterone therapy reduces fertility by negative feedback to the hypothalamus and pituitary, suppressing LH and FSH, thereby inhibiting ovulation and leading to amenorrhea. One study showed that testosterone rapidly induces hypothalamic-pituitary-gonadal suppression and induces anovulation. This study also showed, however, that individuals can break through their hormonal suppression, even after long-term use, and experience dysfunctional ovulation [3] Table 1.

Testosterone Formulations Hembree W. C., Cohen-Kettenis P. T., Gooren L., et al. Endocrine treatment of gender-dysphoric/gender-incongruent persons: an Endocrine Society^*∗*^ clinical practice guideline. J Clin Endocrinol Metab 2017; 102 : 1.

Long-term testosterone therapy has also been associated with altered ovarian histology [4]. One study done in 2010 looked at the ovarian morphology of 112 transgender men who had undergone hysterectomy with bilateral oophorectomy. All had been treated with testosterone therapy for at least 6 months. Evaluation of the ovaries revealed collagenization of the outer cortex, luteinization of stroma cells, stromal hyperplasia, and multifollicular ovaries, typical of polycystic ovarian morphology [5]. Due to these potential effects of GAHT, fertility-preserving measures, such as ovarian tissue cryopreservation, are offered to transgender male patients who desire future pregnancy.

While testosterone has historically been thought to significantly reduce fertility rates, recent studies have shown that conception after receiving long-term exogenous testosterone is possible. A study done in 2019 compared the success of assisted reproductive technology in transgender men who stopped testosterone for 4 months to ciswomen, and showed similar oocyte quality and quantity in both groups after receiving ovarian stimulation [6].

While the effect of long-term testosterone therapy on fertility is not known, transgender men who have received 1 year of testosterone therapy appear to have preserved functional ovarian reserve. These individuals have normal anti-Mullerian hormone levels and preserved antral follicle counts, indicating that they still have ovarian reserve and preserved ovarian function [7].

## 4. Conclusion

Many areas pertaining to the effects of gender-affirming therapy on long-term fertility still require further research. Many studies have shown conflicting data regarding the possibility of pregnancy after receiving gender-affirming therapy. While it was previously thought that conception after receiving hormonal therapy was nearly impossible, many recent studies, including ours, have refuted this claim. Transgender patients should be counseled about fertility-preserving measures prior to hormonal therapy due to the lack of clear data about the effects of long-term hormonal therapy on fertility. New data, however, does provide hope to transgender people who desire biological children and did not undergo fertility preservation therapies prior to starting GAHT. This is exciting and positive news for doctors as well as for patients, as recent data has shown that many transgender individuals do desire to have biological children [7, 8].

Furthermore, increasing evidence that pregnancy is possible in transgender individuals highlights the importance of counseling these patients about contraception to avoid an unwanted pregnancy. Unplanned pregnancies have occurred in transgender male patients, and contraception is often an area overlooked by many health care professionals when treating transgender patients [9].

Our study also highlights the importance of counseling patients on gender-affirming hormone therapy to recognize pregnancy if it occurs, as many medications used to affirm gender have teratogenic side effects. High levels of testosterone during pregnancy have been associated with fetal growth restriction and cardiovascular and metabolic syndrome in the baby and mother [10]. Exogenous testosterone can also be associated with urogenital abnormalities in female fetuses [11]. It is important to discontinue testosterone therapy during pregnancy to avoid these complications.

There has been an increase in studies regarding transgender individuals, with increasing societal acceptance of these individuals. An area that is increasingly being studied and documented in medical literature is transgender pregnancy. Despite this, many areas pertaining to this require further study, such as the long-term effect of testosterone therapy on fertility rates, adverse effects of testosterone on the fetus, and the average time to conception in transgender males.

Very little systemic data exists overall regarding this topic, and further study would not only be greatly beneficial in aiding physicians in providing better care for these patients, but also in aiding patients in making important decisions related to their fertility. This case highlights that transgender male pregnancy is possible and also reflects areas pertaining to this topic that require further study.

## Figures and Tables

**Table 1:** 

Gender-affirming therapy	Dosing Frequency	Advantages	Disadvantages
Intramuscular testosterone	Every 1 or 2 weeks	Better compliance than topical due to less frequent dosage	Fluctuating testosterone levels, injection complications
Subcutaneous testosterone	Bi-weekly or weekly	Better compliance than topical, with a shorter needle than IM forms	Fluctuating testosterone levels, not approved in USA
Testosterone gel	Daily	Ease of application, and less variation in testosterone levels	Slower masculinization than IM or SC, potential transfer with contact
Testosterone patch	Daily	Ease of application, and less variation in testosterone levels	Skin irritation, and lower testosterone levels than gel

## Data Availability

The data used to support the findings of this study are available from the corresponding author upon request.
